# Patients’ Ratings of Family Physician Practices on the Internet: Usage and Associations With Conventional Measures of Quality in the English National Health Service

**DOI:** 10.2196/jmir.2280

**Published:** 2012-10-17

**Authors:** Felix Greaves, Utz J Pape, Henry Lee, Dianna M Smith, Ara Darzi, Azeem Majeed, Christopher Millett

**Affiliations:** ^1^Department of Primary Care and Public HealthImperial College LondonLondonUnited Kingdom; ^2^Centre for Health PolicyInstitute of Global Health InnovationImperial College LondonLondonUnited Kingdom

**Keywords:** Patient Experience, Primary Care, Internet, Quality

## Abstract

**Background:**

Patients are increasingly rating their family physicians on the Internet in the same way as they might rate a hotel on TripAdvisor or a seller on eBay, despite physicians’ concerns about this process.

**Objective:**

This study aims to examine the usage of NHS Choices, a government website that encourages patients to rate the quality of family practices in England, and associations between web-based patient ratings and conventional measures of patient experience and clinical quality in primary care.

**Methods:**

We obtained all (16,952) ratings of family practices posted on NHS Choices between October 2009 and December 2010. We examined associations between patient ratings and family practice and population characteristics. Associations between ratings and survey measures of patient experience and clinical outcomes were examined.

**Results:**

61% of the 8089 family practices in England were rated, and 69% of ratings would recommend their family practice. Practices serving younger, less deprived, and more densely populated areas were more likely to be rated. There were moderate associations with survey measures of patient experience (Spearman ρ 0.37−0.48, *P*<.001 for all 5 variables), but only weak associations with measures of clinical process and outcome (Spearman ρ less than ±0.18, *P*<.001 for 6 of 7 variables).

**Conclusion:**

The frequency of patients rating their family physicians on the Internet is variable in England, but the ratings are generally positive and are moderately associated with other measures of patient experience and weakly associated with clinical quality. Although potentially flawed, patient ratings on the Internet may provide an opportunity for organizational learning and, as it becomes more common, another lens to look at the quality of primary care.

## Introduction

Consumers are using the Internet to rate services and products, for example when they stay in a hotel or buy a product online. This increasingly applies to health care, particularly in the US and UK. A number of websites allowing patients to rate their care have been developed by health care payers and the commercial sector, such as RateMDs or Angie’s List, in an effort to increase transparency and responsiveness of health systems and to help patients choose between providers [1-4].

The value of patient rating websites has been criticized, particularly by family physicians [5], who are concerned that patient ratings are not representative, are unduly negative, may not contain information relevant to the quality of health care provided, and may harm the doctor-patient relationship [6]. However, we also know that physicians may misjudge patients’ care experience and that physician performance can be improved by feedback [7], so these websites may provide useful information for patients and health care workers.

NHS Choices is a government-run website that serves as a comprehensive directory of health care providers in England and includes comparative information on performance of family practices [8]. One of its functions is to allow the public to rate the quality of care they received at their family practice, both by leaving ratings on specific aspects of care and by making comments.

Little is known about who uses patient-rating websites and how unsolicited ratings by patients relate to more conventional measures of patient experience and clinical quality. While some patients may be interested in reviewing other patients’ ratings as a window into family physicians’ interpersonal styles and office amenities, the amount of agreement between these ratings and conventional measures of quality such as patient experience captured in surveys or clinical process and outcome measures will be important in understanding the usefulness of these ratings in quality measurement.

This paper seeks to examine usage patterns of patients’ ratings of family physicians on the Internet. We describe how frequency and nature of rating vary with practice and population characteristics, and we present comparisons between web-based ratings and conventional measures of patient experience and clinical quality.

## Method

### Data Sources and Measures

#### Online Ratings

All patient ratings of family practices posted on the NHS Choices website between October 14, 2009 (the date the function started), and December 31, 2010, were obtained from the Department of Health, aggregated to the practice level. The NHS Choices website allows patients to indicate whether they would recommend a family practice to a friend (yes/no) and rate practices on a scale for four specific domains of quality: whether they were able to get through to the practice by telephone; whether they were involved in decisions about care; whether they were able to get an appointment when they wanted one; and whether they were treated with dignity and respect by staff. Data on the individual characteristics of those leaving ratings online were not available.

#### Traditional Patient Surveys

Survey measures of patient experience were obtained from the national General Practice Patient Survey at the practice level. This is a large mail-based survey sent to 5.7 million patients in 2009/10, with more than 2.1 million responses received (a response rate of 37%) [9].

Population and practice characteristics were obtained from the NHS Information Centre. Practice population variables were: the proportion of patients over 65 years, the index of multiple deprivation (IMD) score (an area-based measure of socioeconomic status including components of income, employment, health, education, crime, and housing), the proportion of the practice population who reported their ethnicity as “white”, the population density of the practice (measured as people per square kilometre), and the practice list size. Variables describing the practice were: whether it was a singlehanded practice, a training practice, and the type of contract it had with the health care payer. The type of contract was listed as either Personal Medical Services (PMS) or General Medical Services (GMS). The GMS contract is nationally agreed, while PMS is locally agreed.

We obtained data on clinical quality of family practices from the NHS Information Centre [10] and from NHS Comparators [11], which are both central repositories of NHS process and outcome data. We selected clinical outcome measures based on three criteria: (1) the measures are commonly used in public reporting, (2) there is known to be variation in practice performance on the measures, and (3) they represented the breadth of activities taking place in family practice. The measures chosen were: proportion of patients with diabetes receiving flu vaccinations, proportion of hypertensive patients with controlled blood pressure (systolic/diastolic less than 150/90 mm Hg), proportion of diabetic patients with controlled HbA1C (less than 7%), percentage of low-cost statin prescribing, cervical screening rate, admission rates for ambulatory care sensitive conditions, and the proportion of achieved clinical Quality and Outcomes Framework (QOF) points from available points. The QOF is the NHS’s pay-for-performance scheme in primary care, which awards points for a variety of clinical activities across acute and chronic disease management and disease prevention.

### Data Linkage

In order to create a complete set of family practices in England, we took the NHS’s list of 8381 practices [12]. This excluded walk-in centers, out-of-hours services and prison health centers. We then excluded 165 practices with a list size less than 1000 and 127 military practices. The total number of practices used in the study is 8089 (96.5% of the total number in England).

In order to link data about the practices from NHS Choices with other practice performance and demographic data, we matched practices by postcode using Excel. We created a computer program that extracted postcodes from the NHS Choices website using Python programming language. This match was checked manually by one person to ensure appropriate linking. Where more than one practice is located at the same postcode, they were manually checked to ensure the correct practice was listed using the names of the practice physicians.

We compared the NHS Choices rating data against patient survey and outcomes data from the 2009/10 financial year. We obtained only incomplete practice demographic descriptive data for 163 practices, but these practices were still included in the analysis as we still had information from the patient survey and NHS Choices. Excluded from this analysis were 24 GP practices (of 5362 with ratings) on NHS Choices that could not be matched to the official list of practices.

### Statistical Analysis

We examined associations between whether a family practice was rated and the practice and population characteristics using logistic multivariate regression. A least square regression analysis was used to examine associations between the proportion of patients willing to recommend the practice and practice and population characteristics.

In order to compare ratings with survey measures of patient experience, we compared ratings for questions on NHS Choices with results of the national General Practice Patient Survey using Spearman rank coefficient of correlation. We selected questions from the national survey that most closely matched the ratings questions on the NHS Choice website. In order to compare ratings with measures of clinical quality, we compared the proportion of people who would recommend the practice on NHS Choices with the traditional quality indicators described above using Spearman rank coefficient of correlation. For ratings on a scale, the mean rating for each practice was calculated. Analysis was done with Stata 11 software.

### Mapping

The level of NHS Choices usage to rate family practices online was mapped by ArcMap 9.3 software, using an Inverse Distance Weighted algorithm. The location of each practice was geocoded [13] and mapped with the corresponding data about ratings usage. Rate of using NHS Choices was measured as the number of ratings or comments divided by practice registered population, expressed as ratings per 1000 people. Where multiple practices share a postcode, the mean value of the rate was used.

## Results

### Descriptive Statistics

**Figure 1:**
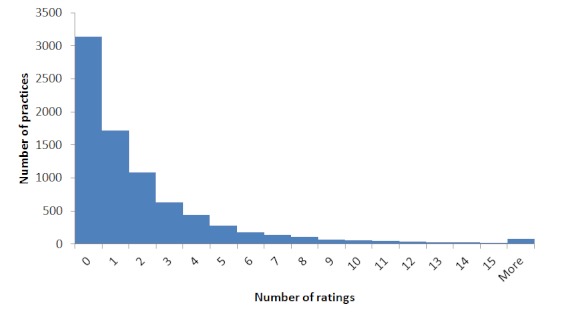
Histogram of rating frequency for all GP practices.

Of the 8089 practices included in the study, 4950 (61%) had been rated on the NHS Choices website. There were 16,952 ratings of these family practices. The mean number of ratings of each practice was 2.1; the median was 1. The range was from 0 to 149 ratings. A histogram of frequency of rating is shown in Figure 1. For those practices that had been rated, average ratings at the practice level for each of the questions by the rating website are shown in Table 1.

**Table 1:** Mean patient ratings posted for questions on NHS Choices.

**Question asked**	**Mean rating**	**Interquartile range**
I would recommend this GP practice to a friend.	64.0 %	33.3–100%
I am able to get through to the practice by telephone.	4.2 out of 5 (1 is lowest, 5 is highest)	3.7–5.0
This GP practice involves me in decisions about my care and treatment.	4.1 out of 5 (1 is lowest, 5 is highest)	3.5–5.0
I am able to get an appointment when I want one.	3.6 out of 5 (1 is lowest, 5 is highest)	3.0–4.3
I am treated with dignity and respect by the staff.	4.1 out of 5 (1 is lowest, 5 is highest)	3.5–5.0

### Associations Between Population and Practice Characteristics and Whether a Practice is Rated

The logistic regression model showed that a larger practice size, a lower proportion of older patients, lower deprivation, higher population density, and not being a singlehanded practice were all positively associated with the likelihood of a practice being rated (Table 2). Whether the practice was a training practice, the proportion of white patients, and the nature of the practice contract did not appear to be associated with being rated. These results remained similar when the nonsignificant variables were consequentially excluded from the model.

**Table 2:** Associations between whether a practice is rated with population and practice characteristics.

**Independent variable**	**Practices that have been rated on NHS Choices**	**Practices that have not been rated on NHS Choices**	**Z statistic**	*P * **value**
Practice population size (Number of registered patients)	7587	5554	15.38	<.001
IMD score of patients (higher is more deprived)	25.1	28.2	-7.82	<.001
Population density (people/km^2^)	458	403	6.72	<.001
Singlehander (% of practices which are singlehanders)	10	20.7	-4.50	<.001
Percentage of population aged over 65 years	15.1	15.6	-3.88	<.001
Percentage of population who are white	87.3	88.3	-1.58	.11
Type of contract (% with PMS contract)	42.7	39.6	-0.71	.48
Training practice (% that are training practices)	32.3	22.0	0.35	.73

### Associations Between Population and Practice Characteristics and the Proportion Recommending

The least square regression model showed that smaller practice size, a higher proportion of white patients, lower population density, lower deprivation, being a training practice, and not being a singlehanded practice were all positively associated with higher levels of recommendation (Table 3). The type of practice contract and the age distribution of the patients were not associated with different recommendation levels. These results remained similar when the nonsignificant variables were consequentially excluded from the model.

**Table 3:** Associations between the proportion of patients recommending a practice with population and practice characteristics.

**Independent variable**	**T statistic**	*P * **value**
Proportion of population who are white	7.39	<.001
Training practice	6.61	<.001
Practice population size	-4.61	<.001
Population density	-4.17	<.001
IMD score of patients	-3.92	<.001
Singlehander	-3.76	<.001
Proportion of population aged over 65 years	1.78	.075
Type of contract	0.62	.53

### Association Between Ratings and Conventional Quality Metrics

Associations between patient ratings and patient experience measures from the family practice survey had Spearman ρ values of between 0.36 and 0.48 and were all significant at the *P*<.001 level (Table 4). A Spearman ρ value of 1 represents a perfect rank correlation, 0 represents no correlation, and -1 represents a perfect negative rank correlation. Comparison between patient ratings and clinical quality indicators showed associations between a better rating on NHS Choices and better quality care for six of the seven variables (*P*<.001); however, Spearman ρ values were all less than ±0.2 (Table 5). There is a very weak negative correlation between ratings and low-cost statin prescriptions.

**Table 4:** Associations between web-based patient ratings and conventional survey measures of patient experience.

**Web-based patient rating**	**NHS General Practice Patient Survey question on patient experience**	**Spearman ρ**	*P * **value**
I would recommend this GP practice to a friend.	Recommending GP practice to someone who has moved to the local area—% yes	0.48	<.001
I am able to get through to the practice by telephone.	Ease of getting through on the phone—% easy	0.43	<.001
This GP practice involves me in decisions about my care and treatment.	Rating of doctor involving you in decisions about your care—% good	0.38	<.001
I am able to get an appointment when I want one.	Able to book ahead for an appointment with a doctor in the past 6 months—% yes	0.37	<.001
I am treated with dignity and respect by the staff.	Rating of doctor treating you with care and concern—Good	0.39	<.001

**Table 5:** Associations between web-based patient ratings of whether the user would recommend the GP practice to a friend and various clinical quality indicators.

**Quality indicator**	**Spearman ρ**	*P * **value**
Proportion of patients with diabetes receiving flu vaccination	0.07	<.001
Controlled blood pressure in hypertensive patients (systolic/diastolic less than 150/90 mm Hg)	0.07	<.001
Controlled HbA1C in patients with diabetes (less than 7%)	0.06	<.001
% low-cost statin prescribing	-0.03	.02
Cervical screening rate	0.18	<.001
Admission rates for ambulatory care sensitive conditions	-0.15	<.001
Total clinical QOF points available	0.11	<.001

### Mapping

**Figure 2:**
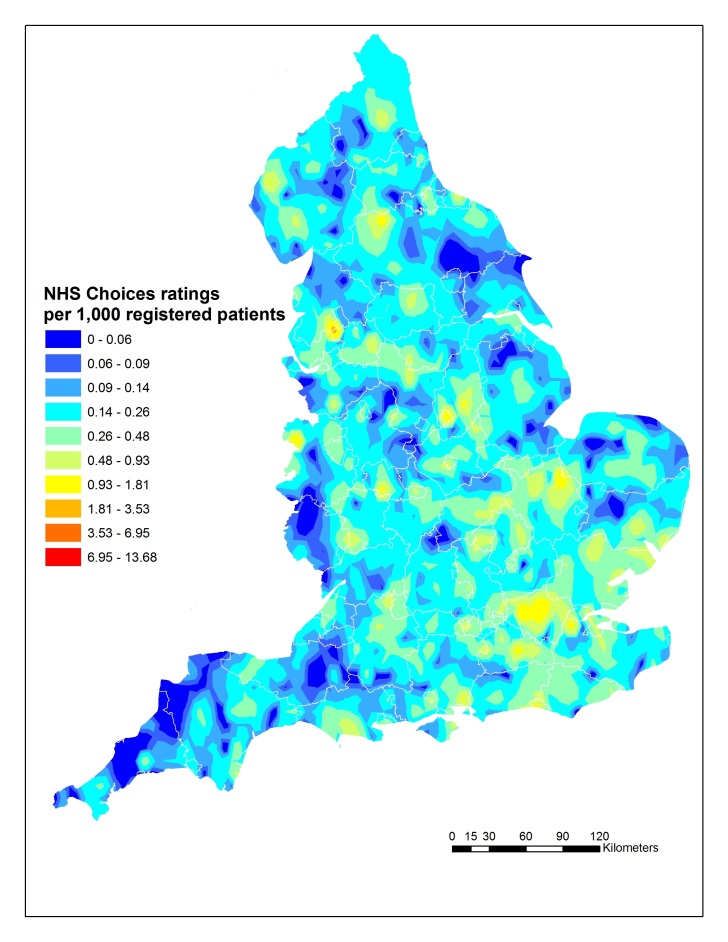
Map of frequency of primary care ratings on the NHS Choices website.

Figure 2 illustrates the spatial variation in the use of online ratings by GP practice. The map indicates that in urban areas usage of NHS Choices tends to be higher, particularly around London. Rates are lower in rural areas, the southwest, and northeast.

## Discussion

The positive nature of responses (with 64% recommending) on the NHS Choices website is in line with other studies of patient-reported Internet feedback in acute hospitals in the UK (where 68% recommend) [14] but is lower than ratings of individual physicians in the USA (88% positive) [1]. These results suggest concerns that online feedback mechanisms will be used only by disgruntled patients hoping to complain are not true. However, as the recommendation level online is lower than in the patient survey (where 82% recommend), the results indicate that there may be a selection bias towards less satisfied patients compared to when patients views are randomly selected. It is also possible that the nature of the NHS Choices website, funded by the government rather than comparable websites in the US that are privately owned, may create a selection bias towards less satisfied patients.

The results demonstrate that usage of patient ratings of family practices via the NHS Choices website has been variable with some practices having more than 100 ratings, but many having none, and an average of only two ratings per practice over the 15-month period covered. A sizable minority (39%) of practices never received a single rating. The data analyzed here represent the initial period of the rating function being available, so usage may well increase as it becomes more popular. However, this usage of online ratings is higher than noted in other settings. A study in Boston, MA, found only 81 of a random sample of 300 physicians had been rated online [1], and a study found only 16% of primary care physicians had been rated on the *RateMDs *website in the US [15]. When compared to the approximately 300 million primary care appointments in England each year [16], the number of responses looks rather small (this corresponds to 0.005% of GP consultations being rated online).

The results suggest that the level of usage of online ratings is different in different communities. Practices serving younger, more urban, and less deprived communities were more likely to be rated. This is in line with previous work showing more usage of the Internet as a health resource in those groups [17].

The demonstration of an association between practice population characteristics and rating usage does not prove a link between individual characteristics and usage; there is a risk of the ecological fallacy, in which an incorrect inference can be drawn about individuals based on aggregated statistics about a group of people. However, these results do confirm that usage rates are variable around the country and suggest that individual characteristics may have a role in influencing usage. Further studies, using individual level data, are required to understand the characteristics of those using ratings websites. The results also show that different practice characteristics are associated with different levels of satisfaction with service, measured as willingness to recommend. This is in line with a wide body of literature on patient experiences, which notes that ethnicity and different socioeconomic factors are associated with satisfaction with medical care [18,19]. These findings may suggest that ratings websites might want to consider ways to broaden their appeal beyond certain groups of users, potentially marketing themselves towards older or rural populations.

Moderate associations between patients’ ratings left on the web and more conventional surveys of patient experiences were found (Spearman ρ values of between 0.38 and 0.48). Due to the large number of practices in the analysis, these associations are all highly significant.

The association between ratings and clinical outcomes is less convincing. Although the results do show increasing levels of recommendation associated with better care across a number of indicators, for many of them the strength of the association is weak and significant only because of the large number of data points. This may be because there is a genuine tension between patient experience and some aspects of technical quality; it is possible to do all of the right things technically in health care and still get a bad outcome. It is also possible that patients’ personal values may differ from the public health perspective captured in the quality metrics. Both the associations with clinical outcomes and survey measures of experience are consistent with similar findings at the hospital level [14,20,21].

These data suggest that we can be more confident in the use of online rating data as a measure of patient experience, despite the many fundamental biases that are an inevitable consequence of this sort of data. However, the extent to which online ratings reflect the technical quality of clinical primary care is less clear. As momentum develops around the need to capture the patient’s experience, these ratings represent a potentially valuable source of information about quality of care when taken with other more conventional measures. Therefore, as such ratings become more common, systems should be developed to allow patients to examine these data alongside other more traditional outcome data, presented in a digestible and accessible way. Similarly, family physicians and practices should develop strategies to respond to these comments in constructive ways [22]. Further research to understand how practices use online feedback to improve the care they provide would be useful. Also, as many practices have few or no ratings online, work is needed to determine the numbers of ratings required over a defined period of time for a patient to obtain a reasonably accurate appreciation of the strengths and weaknesses of a family practice.

### Study Limitations and Strengths

Our study has a number of limitations. We removed practices with a size below 1000 patients, as has been done in other analyses of practice performance [23], as smaller practices are often atypical, such as those recently opened or being closed or serving very specialized populations. However, as these exclusions represent less than 2% of practices in our sample, this is unlikely to have a major bearing on our findings. In addition, the timing of the ratings and the other outcome and experience measures do not match entirely, but we have chosen the available data with the most overlap. The use of practice population characteristics leaves some of our findings prone to the ecological fallacy, in which we make an inference about the nature of individuals based on the characteristics of the aggregated population. In addition, the clinical quality measures used may not reflect true variations in quality between family practices. As with other online rating systems, there is potential for ratings on NHS Choices to be “gamed” by organizations or for fake or multiple entries to be left by individuals. We also note that the some of the outcome metrics chosen here for quality of care are not entirely in the doctor’s hands, but contingent on both the actions of the physician and the patient. This study also found many practices with no or few ratings.

Our study also has strengths. It uses a novel, largely complete national set of online ratings. The unique nature of the NHS’s performance related pay system provides detailed information on clinical performance at the practice level, allowing detailed comparisons to be made.

### Conclusions

These findings represent the first national review of an online ratings system in primary care. Our findings lend support to some of the arguments against online rating systems, particularly that they may have a selection bias. However, they also suggest some positive aspects. They are not just used as a mechanism to complain, as more often than not they are positive, and there are moderate associations with more traditional measures of patient satisfaction. As the numbers of rating per practice are low, they may be less useful as a measure of quality in family practice but they may provide a novel route for organizational learning. However, as numbers of ratings rise, it is likely that they will become a more useful tool for patients to make informed choices about where to receive their care.
